# Delivery Systems of Plasmid DNA and Messenger RNA for Advanced Therapies

**DOI:** 10.3390/pharmaceutics14040810

**Published:** 2022-04-07

**Authors:** Satoshi Uchida

**Affiliations:** 1 Medical Chemistry, Graduate School of Medical Science, Kyoto Prefectural University of Medicine, Kyoto 606-0823, Japan; suchida@koto.kpu-m.ac.jp; 2 Innovation Center of NanoMedicine (iCONM), Kawasaki Institute of Industrial Promotion, Kawasaki 210-0821, Japan

The vast potential of non-viral delivery systems of messenger RNA (mRNA) and plasmid DNA (pDNA) has been demonstrated in the vaccines against coronavirus disease 2019 (COVID-19). Indeed, two formulations of mRNA vaccines from Pfizer–BioNTech and Moderna were approved for emergency use within one year after the pandemic outbreak, and have been administered to billions of people worldwide [1]. Furthermore, a pDNA vaccine developed by Zydus Cadila also obtained emergency approval in India, exhibiting high efficiency for preventing infection in a large clinical trial [2]. These successful examples prompt further research and the development of vaccines and therapeutics based on mRNA and pDNA. The target areas are not limited to preventive vaccination for infectious diseases but expand to therapeutic cancer vaccines, genome editing, and protein replacement therapy.

However, current non-viral systems need improvement. For example, the relatively intense adverse effects of mRNA vaccines, including myocarditis, provoke vaccine hesitancy and debates about repeated boosting. Thus, safer formulations are in demand for mRNA vaccines to become platforms for various infectious diseases. Meanwhile, therapeutic cancer vaccines require more efficient formulations to overcome the immunosuppressive nature of cancers. For other applications, including genome editing and protein replacement therapy, delivery carriers should reach specific tissues and introduce pDNA and mRNA without damaging the tissues. The present Special Issue addresses vigorous efforts to develop mRNA and pDNA delivery systems and apply them to disease treatment to meet these demands.

The development of non-viral delivery systems has two directions. One focuses on the general improvement of delivery processes, which includes preventing extracellular mRNA and pDNA degradation by nucleases, intracellular targeting of mRNA and pDNA to the desired sites, and prolonging the duration of protein expression from mRNA and pDNA. In the other direction, delivery systems are fine-tuned for specific purposes, such as reaching particular tissues and cells to achieve therapeutic goals and stimulating innate immune systems when used in vaccinations.

Several articles and reviews in this Special Issue address the general development of delivery systems. Lipid-based systems are among the most advanced techniques, with two approved mRNA vaccines based on lipid nanoparticles. While numerous reviews focus on lipid-based delivery systems of mRNA and pDNA [3], Delehedde et al. focused on the intracellular processing of LNP mRNA [4]. The cellular uptake pathways, the efficiency of endosomal escape, and the intracellular distribution of mRNA largely influence the protein expression efficiency from mRNA and the therapeutic potentials. Recent advances in the molecular design of LNPs allow the modulation of these processes to maximize the potential of mRNA therapeutics.

LNPs possess strong immunostimulating properties and liver tropisms [5,6], which may cause problems in applications other than vaccinations and the treatment of liver diseases. These issues have motivated many researchers to pursue other delivery options. Cell-penetrating peptides (CPPs) have several features distinct from LNPs, and Yokoo et al. reviewed this emerging field [7]. CCPs can work as a ligand to target specific cells, modulate cellular uptake pathways, and protect mRNA from degradation in the cytosol. Strikingly, CPPs from arginines and α-aminoisobutyric acid (Aib), the simplest form of an α,α-disubstituted amino acid, were stably bound to mRNA in the cytosol for mRNA protection, allowing for prolonged protein expression from mRNA at least for three days [8]. However, obtaining this functionality using lipids is challenging because lipids are likely to integrate into membrane components and release mRNA after cellular uptake.

Nasr et al. developed an elaborate design for the co-delivery of mRNA and pDNA [9]. As these two nucleic acid species exhibit different temporal protein expression profiles, their co-delivery allows the sequential expression of proteins. They first prepared the core from gelatin and pDNA and coated it sequentially with cationic peptides and mRNA. Their system provided more efficient protein expression from mRNA and pDNA than commercial lipid and polymer transfection reagents.

Both cationic lipids and polymers have a toxicity issue, and thus, several clinical trials of mRNA therapeutics used naked mRNA [10,11,12]. However, naked mRNA was susceptible to nuclease attack, requiring systems for improving nuclease stability without cationic lipids and polymers. Yoshinaga et al. proposed mRNA PEGylation by hybridizing mRNA with PEGylated complementary RNA oligonucleotides [13]. PEGylation improved nuclease stability in a test tube. Notably, the translational activity of mRNA was preserved even after PEGylation, presumably because PEGylated RNA oligonucleotide may be detached from mRNA selectively in the cytosol during the process of translation [14,15].

Minicircle DNA is a robust technique to improve the efficiency of pDNA delivery. Minicircles are prepared by removing bacterial backbones from pDNA by recombination. Their small size is favorable for efficient pDNA introduction. While this technique is versatile for various biomedical purposes, Rim et al. reviewed its application to cartilage diseases [16]. The introduction of anti-inflammatory and chondrogenic genes has promise for treating osteoarthritis and rheumatoid arthritis, which are intractable due to the limited regenerative capacity of cartilage. Interestingly, the minicircle technique has also been used for the basic research of cartilage diseases and the preparation of disease models.

Alongside improving the general performance of mRNA and pDNA, fine-tuning the delivery systems for specific therapeutic purposes is critical. In many therapeutic settings, efficient and selective delivery of mRNA and pDNA to particular cells and tissues is necessary. Ligands’ introduction to delivery carriers is a well-established method of targeting. Serra et al. utilized mannose ligands for delivering minicircle DNA to macrophages for future application in DNA vaccines [17]. Singh et al. equipped selenium-based mRNA nanoparticles with lactobionic acid, a ligand of the asialoglycoprotein receptor, for targeting hepatocellular carcinoma [18]. Notably, the compositions of delivery nanoparticles were optimized to maximize the ligands’ functionalities in both cases.

Modulating the physicochemical characteristics of mRNA and pDNA carriers provides another option for tissue targeting. For example, negatively charged mRNA lipoplexes selectively accumulated to the spleen after systemic injection, providing an excellent platform for cancer vaccination [19]. Indeed, one such system demonstrated promising outcomes in a clinical trial of mRNA-based vaccination for melanoma [20]. Tusup et al. employed this system for mRNA cancer vaccines targeting CDR3 hypervariable regions of T cell receptors [21]. This region undergoes gene recombination during T cell development and thus can be considered a neo-antigen, an effective target of cancer vaccines. Experiments using a mouse model successfully provided proof of concept for this strategy.

The brain is among the most challenging organs for delivering mRNA and pDNA, as the blood-brain barrier (BBB) inhibits the transport of materials from the blood to the brain. One approach to brain targeting is introducing ligands onto delivery carriers, such as transferrin, to facilitate the transport across the BBB. Alternatively, the BBB can be bypassed by injection to the brain parenchyma or cerebrospinal fluids. Hauck et al. reviewed this research field [22].

Only a small number of organs, such as the liver and spleen, are effectively targeted by the current delivery systems of mRNA and pDNA. Meanwhile, numerous diseases require the expression of therapeutic proteins in non-targetable organs. For example, Fabry disease, caused by the deficiency of a lysosomal enzyme, α-galactosidase A (α-Gal A), results in the deposition of glycosphingolipids in vascular endothelial and smooth muscle cells throughout the body. Notably, α-Gal A is secreted systemically once expressed in some organs. Rodriguez-Castejon et al. utilized this property to introduce α-Gal A pDNA to the liver using LNPs and successfully treated a mouse model of Fabry disease [23]. Similarly, the LNP-based delivery of mRNA and pDNA to the liver is often employed to systemically supply secreted proteins [24].

Vaccination requires unique designs for mRNA and pDNA delivery systems to simultaneously induce proper immunostimulation and efficient antigen expression. Complexes from protamine and mRNA possess these dual functions. Jarzebska et al. reviewed the research on this system, including its clinical application to infectious disease prevention and cancer immunotherapy [25]. By incorporating the functionalities of immunostimulatory adjuvants into the delivery material, this strategy minimizes the components for mRNA vaccines. Such minimalistic approaches were employed in other types of nucleic acid vaccines, including those based on LNPs, as reviewed by Abbasi et al. [26]. Notably, the immune receptors responsible for the adjuvant effects have been discovered in many vaccines.

In addition to the in vivo delivery of mRNA and pDNA, the combination of their ex vivo delivery with cell transplantation therapy has garnered much attention. In many clinical trials, mRNA electroporation was used to introduce tumor-associated antigens into dendritic cells and chimeric antigen receptors or T cell receptors into T cells for cancer immunotherapy. Campillo-Davo et al. comprehensively reviewed this topic, from the physics and biology of electroporation to mRNA production and electroporation in clinical settings [27].

The present Special Issue provides comprehensive reviews of non-viral mRNA and pDNA delivery by addressing the various delivery technologies of mRNA and pDNA, from basic research to therapeutic and clinical application.
